# *Treponema pallidum* membrane protein Tp47 induced autophagy and inhibited cell migration in HMC3 cells via the PI3K/AKT/FOXO1 pathway

**DOI:** 10.1111/jcmm.17872

**Published:** 2023-07-24

**Authors:** Lin Xie, Wei Li, Xin‐Qi Zheng, Li‐Li Liu, Li‐Rong Lin, Jian‐Jun Niu, Tian‐Ci Yang

**Affiliations:** ^1^ Center of Clinical Laboratory, Zhongshan Hospital of Xiamen University, School of Medicine Xiamen University Xiamen China; ^2^ Institute of Infectious Disease, School of Medicine Xiamen University Xiamen China

**Keywords:** autophagy, migration, PI3K/AKT/FOXO1, *Treponema pallidum*, *Treponema pallidum* membrane protein Tp47

## Abstract

The migratory ability of microglia facilitates their rapid transport to a site of injury to kill and remove pathogens. However, the effect of *Treponema pallidum* membrane proteins on microglia migration remains unclear. The effect of Tp47 on the migration ability and autophagy and related mechanisms were investigated using the human microglial clone 3 cell line. Tp47 inhibited microglia migration, the expression of autophagy‐associated protein P62 decreased, the expression of Beclin‐1 and LC3‐II/LC3‐I increased, and the autophagic flux increased in this process. Furthermore, autophagy was significantly inhibited, and microglial cell migration was significantly increased after neutralisation with an anti‐Tp47 antibody. In addition, Tp47 significantly inhibited the expression of p‐PI3K, p‐AKT, and p‐mTOR proteins, and the sequential activation of steps in the PI3K/AKT/mTOR pathways effectively prevented Tp47‐induced autophagy. Moreover, Tp47 significantly inhibited the expression of p‐FOXO1 protein and promoted FOXO1 nuclear translocation. Inhibition of FOXO1 effectively suppressed Tp47‐induced activation of autophagy and inhibition of migration. *Treponema pallidum* membrane protein Tp47‐induced autophagy and inhibited cell migration in HMC3 Cells via the PI3K/AKT/FOXO1 pathway. These data will contribute to understanding the mechanism by which *T. pallidum* escapes immune killing and clearance after invasion into the central nervous system.

## 1 INTRODUCTION

Neurosyphilis is a disease of the nervous system caused by *Treponema pallidum*.^1^ However, the pathogenesis of neurosyphilis remains unknown. Microglia are macrophages of the central nervous system. The physiological function of macrophages is critically important in the body's responses to bacteria, as they are the first line of defence against invading microorganisms. Their efficacy in containing and eliminating bacteria is critical for assessing the risk of a patient becoming infected.^2^ Dysfunction of microglial cells prevents them from accurately migrating to the site of injury to kill and remove pathogens.^3^ Therefore, studying the changes in the migration ability of microglial cells will contribute to understanding the pathogenesis of neurosyphilis.

Our previous research revealed that the *T. pallidum* membrane protein Tp47 promoted microglial autophagy.^4^ Autophagy is a highly preserved process of self‐digestion involving the breakdown of organelles, protein clusters and cytoplasmic proteins by lysosomes.^5^ Research has suggested that selective autophagy plays a major role in the dynamic turnover of integrin‐based focal adhesion sites during cell motility, although the exact molecular mechanisms underlying this process are still being investigated.^6^ Therefore, autophagy has been described as a regulator of cell migration. PI3K/AKT/mTOR is a classic autophagy signalling pathway.^7^ Autophagy involves signalling, autophagy movement and vesicle fusion, all related to the PI3K/AKT signalling pathway.^8^ AKT activation controls apoptosis, autophagy and development through the phosphorylation of downstream proteins such as mammalian target of rapamycin (mTOR) kinase and forkhead box protein O1 (FOXO1) transcription factor.^9^

To explore the involvement of Tp47 in microglial migration and its underlying mechanisms, the impact of Tp47 on autophagy and microglial migration was systematically explored using the human microglial clone 3 (HMC3) cell line in this study.

## 2 MATERIALS AND METHODS

### 2.1 Preparation of recombinant *Treponema pallidum* Tp47

Recombinant Tp47 was isolated, and endotoxin was eliminated using an established method.^10^ The tachypleus amebocyte lysate test (Chinese Horseshoe Crab Reagent Manufactory, Ltd.) revealed that the endotoxin contamination of Tp47 was less than 0.05 endotoxin units.

### 2.2 Cell culture

HMC3 cells were acquired from the American Type Culture Collection (Manassas, USA). The HMC3 cells were maintained in a specialized medium (Procell Life Science & Technology Co., Ltd.) at 37°C under a 5% CO_2_ atmosphere. This study was approved by the Institutional Ethics Committee of Zhongshan Hospital of Xiamen University, China.

### 2.3 Transwell migration assay

Transwell migration assays were conducted using Corning Transwell chambers (#3422, Corning).^11^ Medium (0.6 mL) with 10% foetal bovine serum was added to the lower chamber of the transwell, and 0.1 mL of cells diluted with serum‐free medium and different concentrations of Tp47 or inhibitors were placed in the upper chamber. Cells were fixed with 4% polymethanol solution for 15 min and stained with 0.1% crystal violet for 20 min.

### 2.4 Western blotting

Western blotting was performed as described previously.^4^ Antibodies against the following proteins were used: PI3K (1:1000, 4249S, Cell Signaling Technology), p‐PI3K (1:1000, 13857S, Cell Signaling Technology), AKT (1:1000, 9272S, Cell Signaling Technology), p‐AKT (1:1000, 9271T, Cell Signaling Technology), mTOR (1:1000, 2972S, Cell Signaling Technology), p‐mTOR (1:1000, 2971S, Cell Signaling Technology), FOXO1 (1:1000, 2880T, Cell Signaling Technology), p‐FOXO1 (1:1000, 9461T, Cell Signaling Technology), GAPDH (1:5000, 60004‐1‐Ig, Proteintech), SQSTM1/p62 (1:1000, ab109012, Abcam), LC3B (1:1000, ab51520, Abcam) and Beclin‐1 (1:1000, ab210498, Abcam). Optical density was assessed by ImageJ software (National Institutes of Health). Protein bands were adjusted to the GAPDH expression in the same sample.

### 2.5 mRFP‐GFP‐LC3 adenovirus vector infection

Autophagosome–lysosome fusion was investigated using mRFP‐GFP‐LC3 (HB‐AP210 0001, HANBIO) according to a previously described protocol.^12^ HMC3 cells were transfected with an adenovirus expressing a plasmid featuring tandem mRFP‐GFP‐LC3 fluorescent proteins, and the cells were treated with Tp47 alone or in combination with the FOXO1 inhibitor AS1842856 for a period of 24 h. A confocal laser scanning microscope was used to observe the fluorescence images (LSM780, Zeiss).

### 2.6 Immunofluorescence staining

Immunofluorescence staining was performed as described previously.^13^ A primary antibody against the target protein FOXO1 (1:200, 2880T, Cell Signaling Technology) and Alexa Fluor® 488‐conjugated secondary antibody (1:1000, ab150077, Abcam) were used. A confocal laser scanning microscope was used to visualize the fluorescence images (LSM780, Zeiss).

### 2.7 Extraction of nuclear and cytoplasmic proteins and western blotting

Total protein was prepared from cultured HMC3 cells. Nuclear and cytoplasmic proteins were extracted using nuclear and cytoplasmic extraction reagents (#78833, Thermo Fisher Scientific) as previously described.^14^ The isolated nucleoprotein and cytoplasmic protein samples were analysed by western blotting. Immunoblotting was performed with primary antibodies against FOXO1 (1:200, 2880T, Cell Signaling Technology). GAPDH (1:5000, ab8245, Abcam) and Lamin B (1:1000, ab16048, Abcam) served as internal controls for detecting the expression levels in cytoplasmic protein lysates and nuclear protein lysates, respectively.

### 2.8 Statistical analysis

All experiments were repeated at least three times, and the data are presented as the mean ± SD. GraphPad Prism 9.0 (GraphPad Software) was utilized to perform all statistical analyses. Student's *t‐*test was used to compare with two groups. One‐way anova was utilized to compare group means with one independent variable, while two‐way anova was utilized to compare group means with two independent variables. Following anova, Tukey's test was utilized for post hoc comparisons. A *p* value <0.05 was regarded statistically significant.

## 3 RESULTS

### 3.1 Tp47 impaired HMC3 cell migration

**FIGURE 1:**
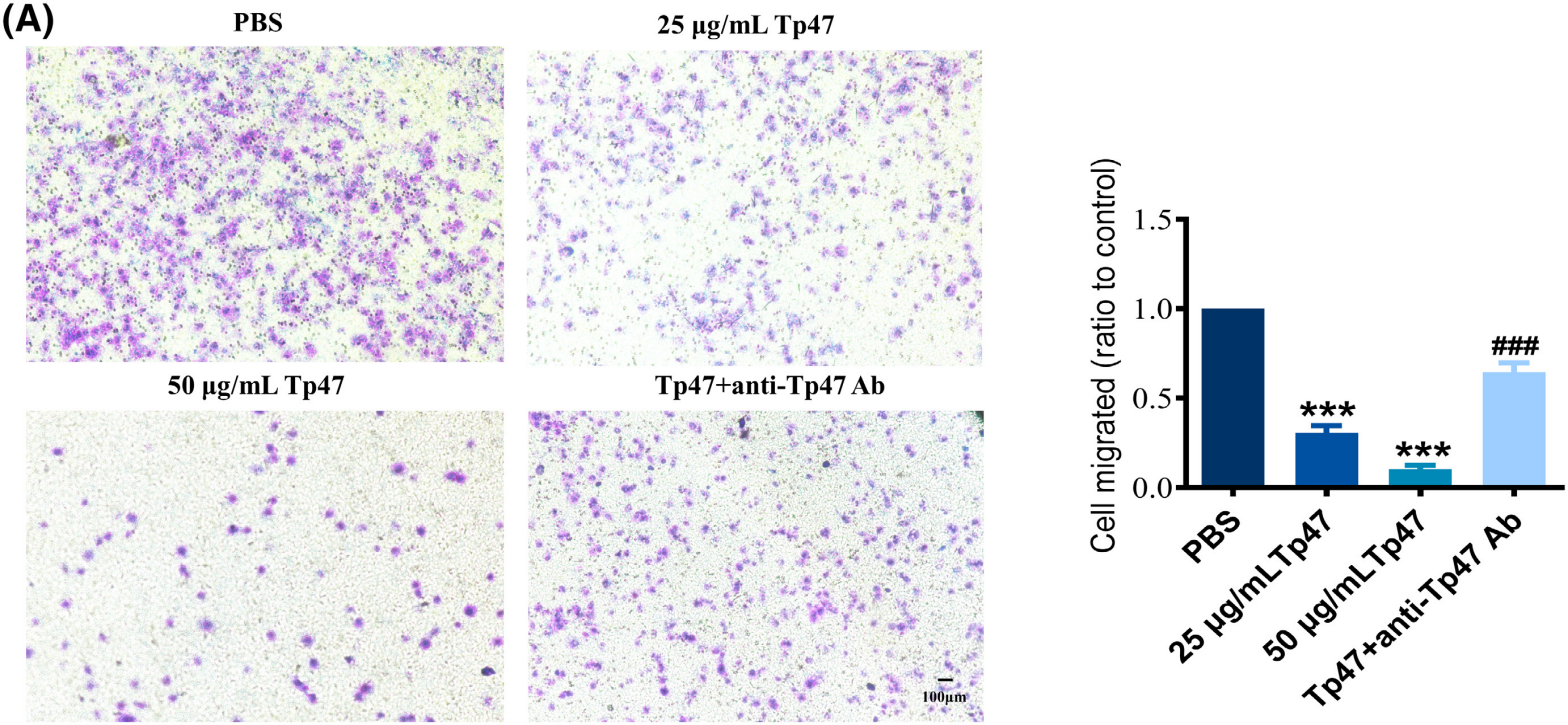
(A) Tp47 impaired HMC3 cell migration. One‐way anova was used to compare three or more group means with one independent variable. All data were normalized to the PBS group and are presented as the means ± SD. *p versus PBS, ***p < 0.001. ^#^ *p* versus Tp47, ^###^ *p* < 0.001. Scale bars = 100 μm. Abbreviations: anti‐Tp47 Ab, anti‐Tp47 antibody; PBS, phosphate‐buffered saline.

HMC3 cells were treated with Tp47 (25 and 50 μg/mL) for 24 h, and the effect on vertical migration of HMC3 cells increased by Tp47 was evaluated with a transwell migration assay. Tp47 significantly impaired the migration of HMC3 cells. The cell migration rate was significantly reduced by 25 μg/mL Tp47 (*p* < 0.001) and the minimal migration rate was detected at 50 μg/mL Tp47 (*p* < 0.001). Additionally, an anti‐Tp47 antibody was incubated with 25 μg/mL Tp47 in the above experiment, and the results showed that the anti‐Tp47 antibody could restore HMC3 cell migration capacity that was resulted from Tp47 (*p* < 0.001; Figure 1A).

### 3.2 Tp47‐mediated autophagy modulates cell migration in HMC3 cells

**FIGURE 2:**
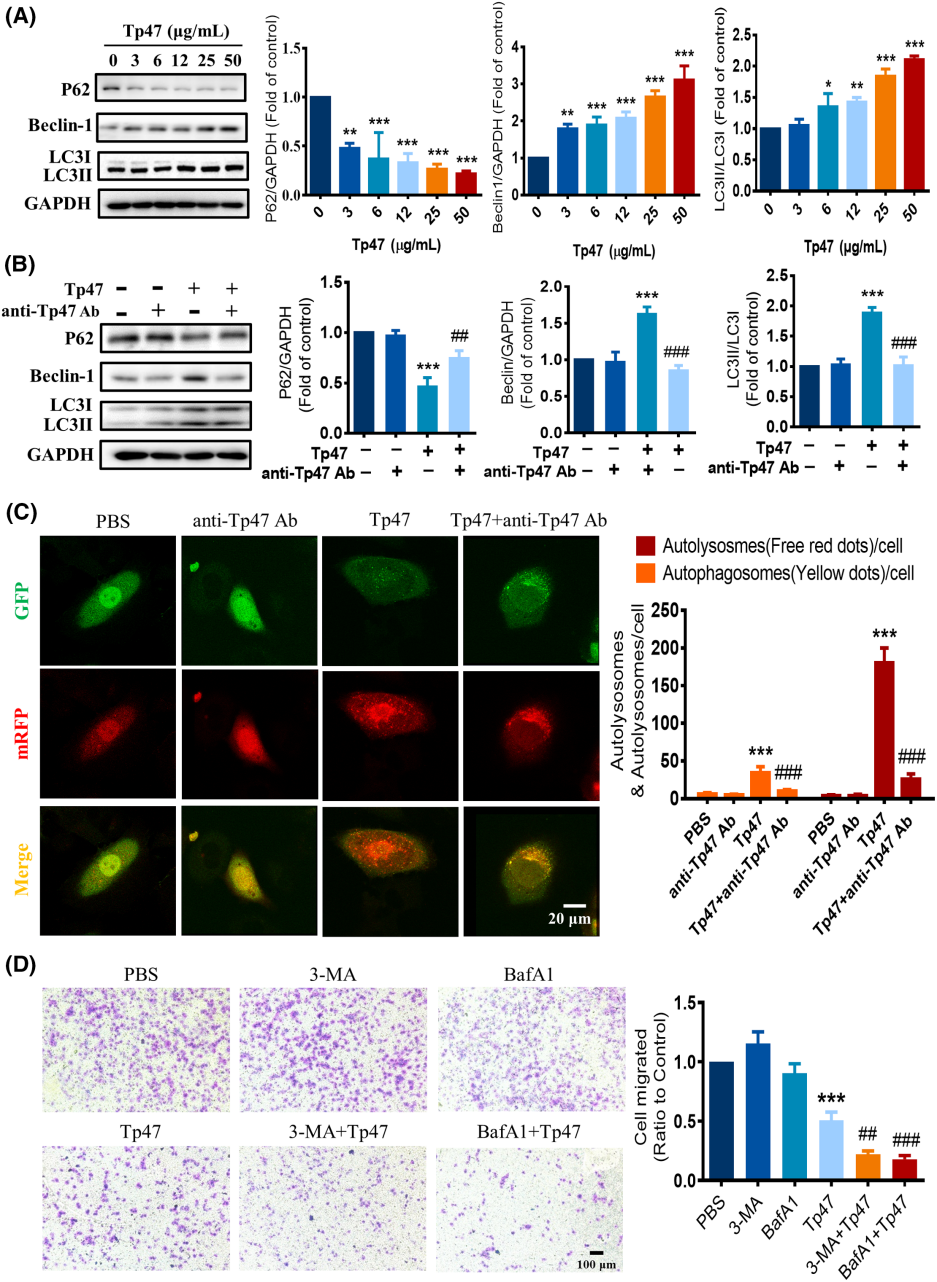
Tp47 induced autophagy to impair HMC3 cell migration. (A) The expression of autophagy‐related proteins in HMC3 cells treated with varying concentrations of Tp47. (B) The effect of an anti‐Tp47 antibody on the expression of autophagy‐related proteins. (C) The effect of an anti‐Tp47 antibody on autophagic flux. Scale bars = 20 μm. (D) The effect of the autophagy inhibitors 3‐MA and BafA1 on HMC3 cell migration. Scale bars = 100 μm. All data were normalized to the PBS group and are presented as the means ± SD. One‐way anova was utilized to compare multiple groups means with one independent variable. Two‐way anova was employed to compare multiple groups means with two independent variables. Post hoc comparisons were conducted using Tukey's *t*‐test. *p versus PBS, *p < 0.05, **p < 0.01 and ***p < 0.001. ^#^ *p* versus Tp47, ^##^ *p* < 0.01, ^###^ *P* < 0.001. Abbreviations: 3‐MA, 3‐methyladenine; anti‐Tp47 Ab, anti‐Tp47 antibody; BafA1, bafilomycin A1; PBS, phosphate‐buffered saline.

To assess the impact of Tp47 on migration through autophagy, HMC3 cells were incubated with 0, 3, 6, 12, 25 and 50 μg/mL of Tp47 for 24 h to examine the influence of Tp47 on migration through autophagy. The expression of autophagy‐related proteins depending on the concentration of Tp47 is shown in Figure 2A. The protein expression of P62 was found to be significantly reduced in response to Tp47 in a dose‐dependent manner, with a decrease as low as 50 μg/mL (*p* < 0.001). In contrast, the protein expression of Beclin‐1 was found to be significantly increased in response to Tp47 in a dose‐dependent manner, with the highest response observed at 50 μg/mL (*p* < 0.001) and the LC3II/LC3I ratio increased significantly in response to Tp47, reaching its peak at 50 μg/mL (*p* < 0.001). In addition, the anti‐Tp47 antibody inhibited the degradation of P62 (*p* < 0.01), the elevation of Beclin‐1 expression (*p* < 0.001) and the blocked conversion of LC3I to LC3II (*p* < 0.001) caused by 25 μg/mL Tp47 in the above experiment (Figure 2B). Moreover, to further examine the effect of Tp47 on autophagic flow, the mRFP‐GFP‐LC3 adenovirus was used. As shown in Figure 2C, the cells stimulated with Tp47 contained significantly more intense red puncta (signifying autolysosomes) than the PBS group (*p* < 0.001), revealing that Tp47 promoted autophagic flux. Furthermore, an anti‐Tp47 antibody was used to explore the phenomenon of autophagic flow blockade (*p* < 0.001). To further assess the impact of autophagy on HMC3 cell migration, HMC3 cells were pretreated with 3‐MA and BafA1 inhibitors, which target various stages of autophagy. The results demonstrated that 3‐MA and BafA1 significantly decreased the cell migration induced by Tp47 (*p* < 0.01 and *p* < 0.001; Figure 2D).

### 3.3 Tp47 induced autophagy by suppressing the PI3K/AKT/mTOR pathway in HMC3 cells

**FIGURE 3:**
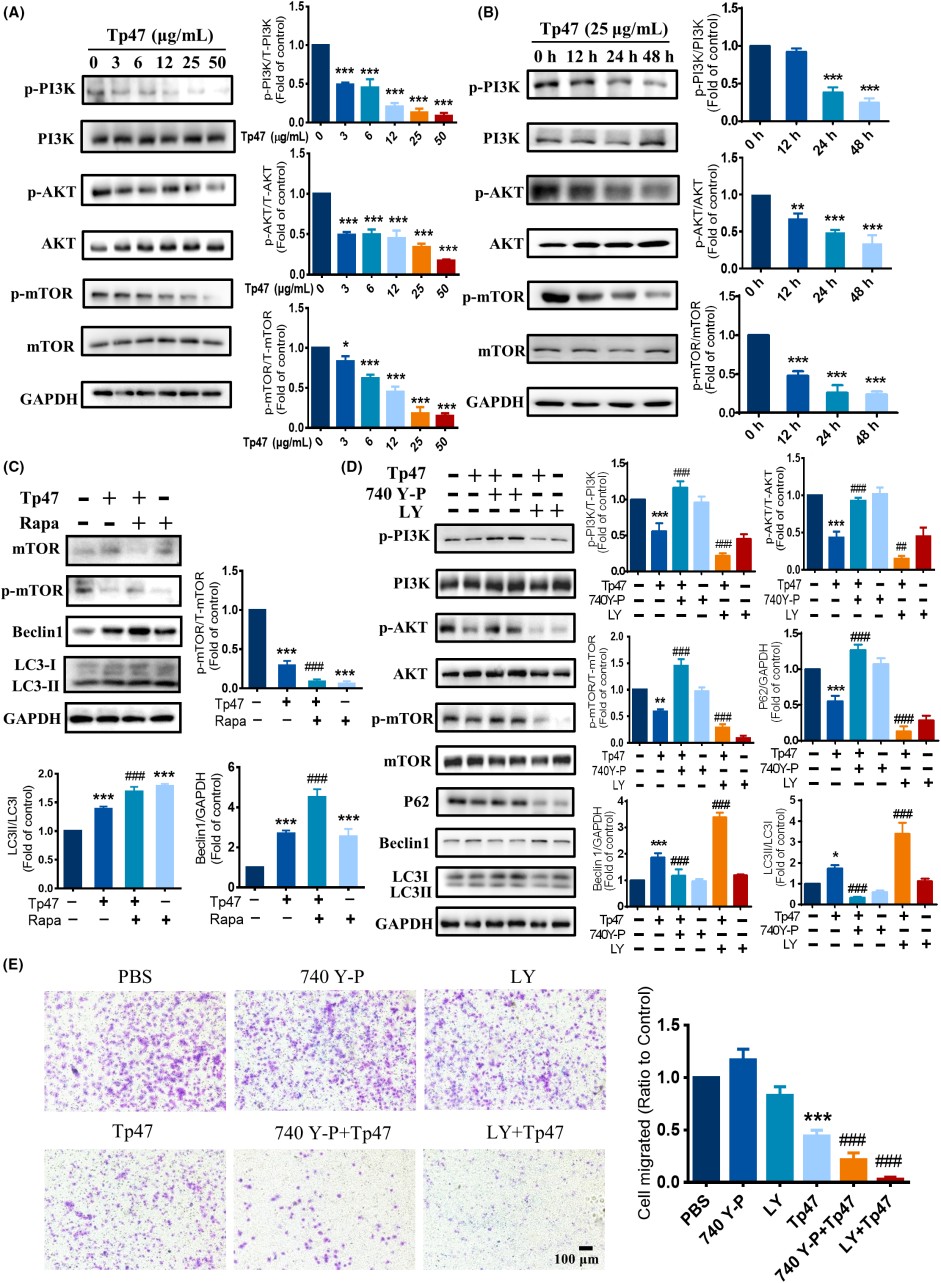
Tp47 induced autophagy by suppressing the PI3K/AKT/mTOR pathway in HMC3 cells. (A) The protein levels of p‐PI3K, p‐AKT and p‐mTOR in HMC3 cells treated with various concentrations of Tp47. (B) The protein levels of p‐PI3K, p‐AKT and p‐mTOR in HMC3 cells treated with Tp47 for different times. (C) The effect of rapamycin on the expression levels autophagy‐related proteins. (D) The effect of PI3K/AKT inhibition on the expression levels of mTOR and autophagy‐related proteins. (E) The effect of PI3K/AKT inhibition on HMC3 cell migration. Scale bars = 100 μm. All data were normalized to the PBS group and are presented as the means ± SD. One‐way anova was utilized to compare multiple groups means with one independent variable. *p versus PBS, *p < 0.05, **p < 0.01 and ***p < 0.001. ^#^ *p* versus Tp47, ^##^ *p* < 0.01, ^###^ *p* < 0.001. Abbreviations: LY, LY294002; PBS, phosphate‐buffered saline; Rapa, rapamycin.

Evidence has shown that the PI3K/AKT/mTOR pathway may affect autophagy.^15^ Western blotting was used to examine the impact of the PI3K/AKT/mTOR pathway on Tp47‐induced autophagy of HMC3 cells. As shown in Figure 3A, the protein expression levels of p‐PI3K and p‐AKT were significantly increased in response to 3 μg/mL Tp47 (*p* < 0.001) and attained the lowest response level with 50 μg/mL Tp47 (*p* < 0.001). Additionally, the protein expression levels of p‐mTOR presented similar results. In response to the incubation of HMC3 cells with 25 μg/mL Tp47 for 0, 12, 24 and 48 h, PI3K/AKT/mTOR pathway phosphorylation was stimulated by Tp47 in a time‐dependent manner. Tp47 significantly suppressed p‐PI3K, p‐AKT and p‐mTOR expression at 24 and 48 h (*p* < 0.001 and *p* < 0.001; Figure 3B). To further explore the role of mTOR in Tp47‐induced autophagy, HMC3 cells were pretreated with the mTOR inhibitor rapamycin and then incubated with 25 μg/mL Tp47 for 24 h. Rapamycin further increased the protein expression levels of Beclin‐1 (*p* < 0.001) and the conversion of LC3I to LC3II (*p* < 0.001; Figure 3C). To reveal the role of the PI3K/AKT/mTOR signalling pathway in Tp47‐induced autophagy, the PI3K/AKT activator 740 Y‐P and the PI3K/AKT inhibitor LY294002 were used. The results demonstrated that 740 Y‐P significantly improved the protein expression of p‐PI3K, p‐AKT and p‐mTOR reduced by Tp47 (*p* < 0.001) and inhibited the degradation of P62 (*p* < 0.001), the expression of Beclin‐1 (*p* < 0.001), and the change of LC3I to LC3II (*p* < 0.001) caused by Tp47 (Figure 3D). In addition, LY294002 significantly diminished the protein expression of p‐PI3K, p‐AKT and p‐mTOR decreased by Tp47 (*p* < 0.01) and promoted the degradation of P62 (*p* < 0.001), the expression of Beclin‐1 (*p* < 0.001), and the conversion of LC3I to LC3II (*p* < 0.001) caused by Tp47 (Figure 3D). The role of the PI3K/AKT/mTOR signalling pathway in HMC3 cell migration was further explored. As shown in Figure 3E, 740 Y‐P improved Tp47‐induced cell migration inhibition (*p* < 0.001), and LY294002 further decreased Tp47‐induced cell migration inhibition (*p* < 0.001). Together, these results suggest that Tp47 induced autophagy by suppressing the PI3K/AKT/mTOR pathway to impair migration in HMC3 cells.

### 3.4 Tp47 promoted FOXO1 transcriptional activation

**FIGURE 4:**
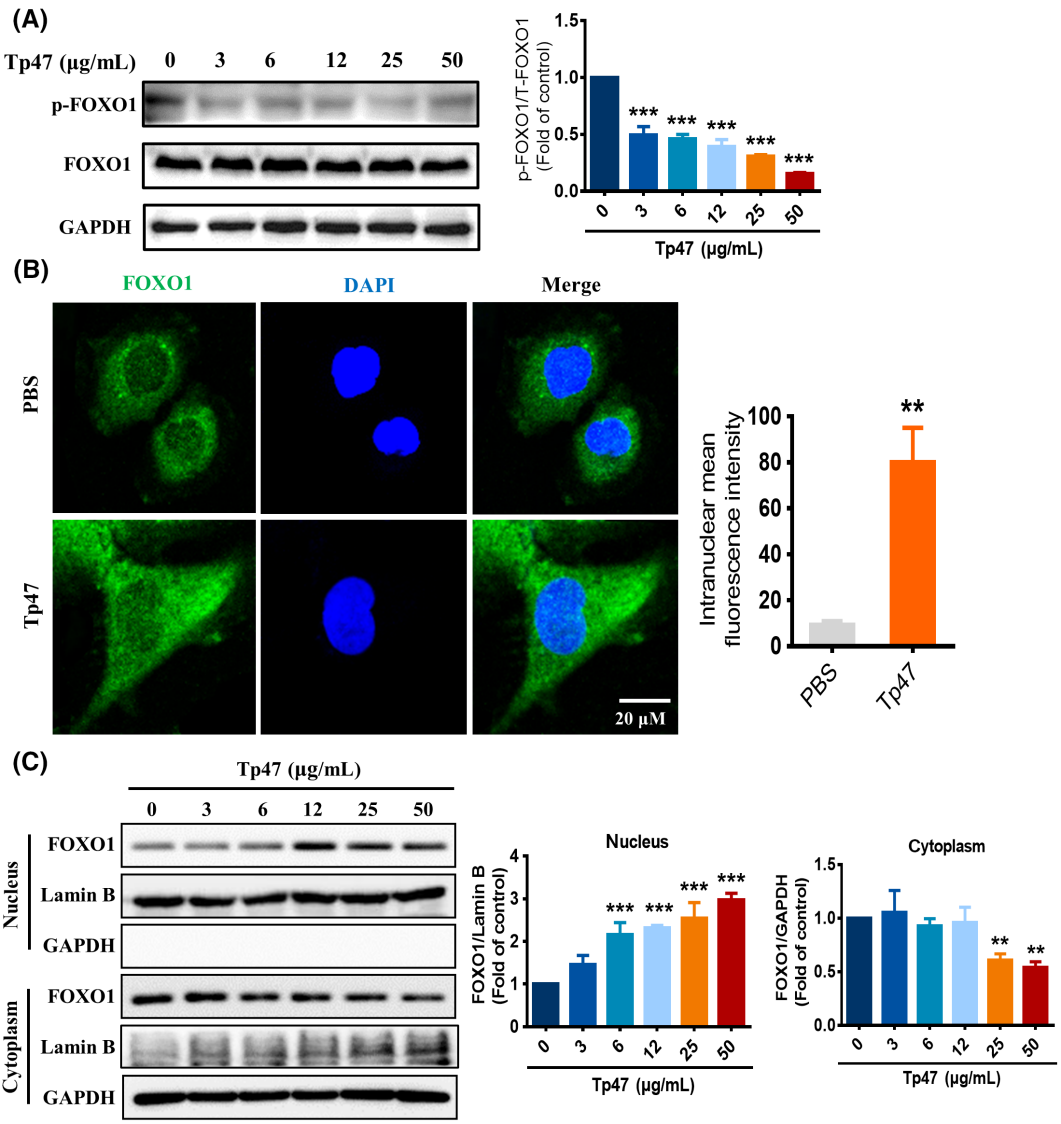
Tp47 promoted FOXO1 transcriptional activation. (A) The effects of different concentrations of Tp47 on p‐FOXO1 protein expression. (B) Analysis of nuclear translocation of FOXO1 by immunofluorescence. Scale bar = 20 μm. (C) The expression levels of FOXO1 in cytoplasmic fractions and nuclear extracts determined by western blotting. All data are presented as the means ± SDs. One‐way anova was used to compare three or more group means with one independent variable. Student's *t*‐test was used to compare two groups. *p versus PBS, **p < 0.01, and ***p < 0.001. Abbreviations: PBS, phosphate‐buffered saline.

FOXO1 nuclear exclusion and inactivity is due to the phosphorylation of FOXO1 mediated by PI3K/AKT pathway activation.^16^ To investigate whether Tp47 affects the nuclear translocation of FOXO1, the protein expression of p‐FOXO1 was measured by western blotting. HMC3 cells were treated with Tp47 (0, 3, 6, 12, 25 and 50 μg/mL) for 24 h. The protein expression of p‐FOXO1 was stimulated by Tp47 in a concentration‐dependent manner (Figure 4A). Compared to cells in the control group, the protein expression of p‐FOXO1 were significantly decreased in response to 3 μg/mL Tp47 (*p* < 0.001) and decreased to its lowest response level with 50 μg/mL Tp47 (*p* < 0.001). Furthermore, nuclear translocation of FOXO1 was observed by immunofluorescence analysis, and cells stimulated with 25 μg/mL Tp47 presented more green puncta in the nucleus than cells in the PBS group (*p* < 0.01; Figure 4B). To further confirm the nuclear translocation of FOXO1, the levels of FOXO1 in cytoplasmic fractions and in nuclear extracts were determined by western blotting. After treatment with Tp47, the protein expression levels of FOXO1 in nuclear extracts significantly increased in a concentration‐dependent manner (*p* < 0.001), and the protein expression of FOXO1 in the cytoplasm significantly decreased (*p* < 0.01; Figure 4C). These results demonstrated that Tp47 promoted FOXO1 transcriptional activation.

### 3.5 PI3K/AKT promoted Tp47‐induced autophagy through FOXO1

**FIGURE 5:**
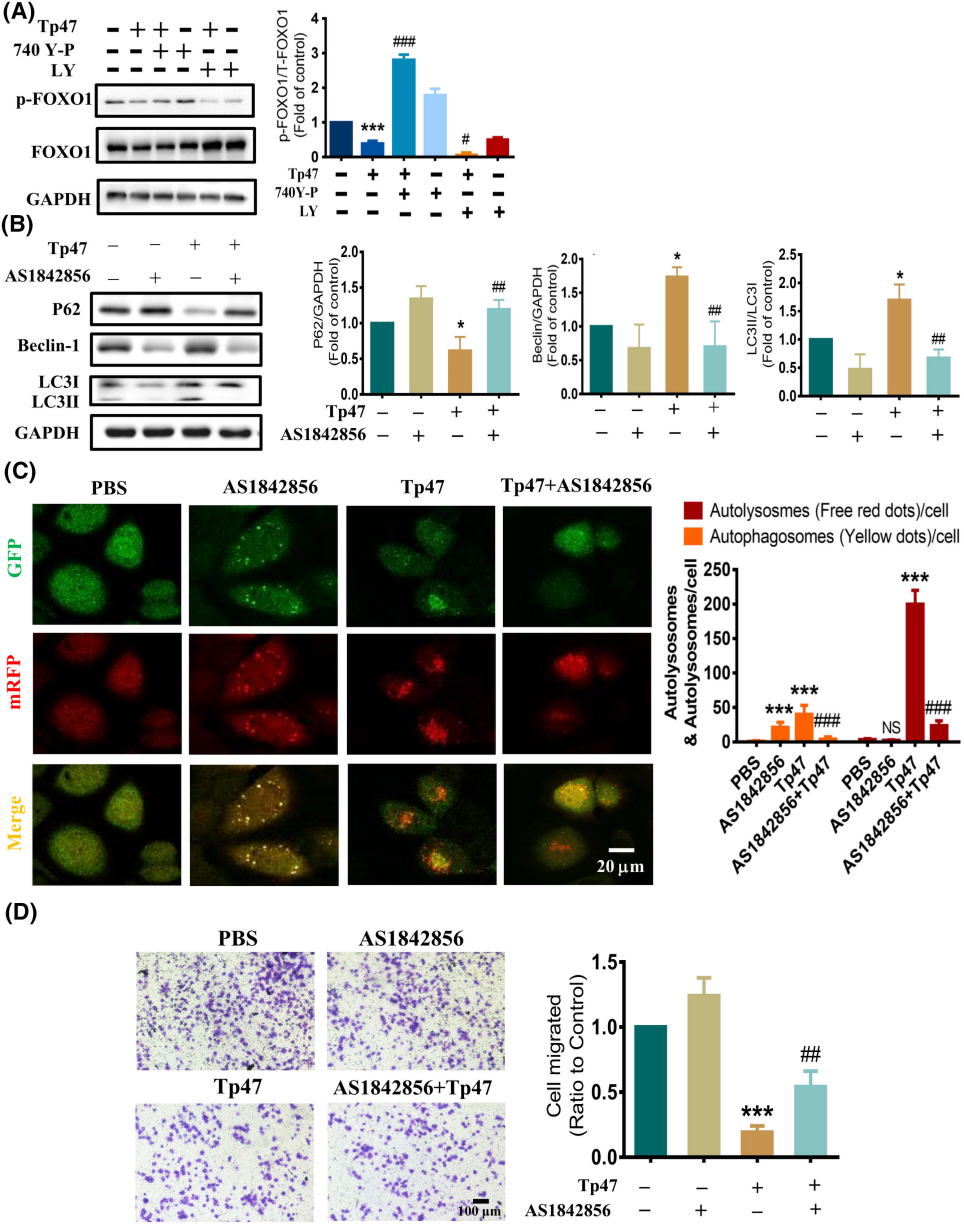
PI3K/AKT promoted Tp47‐induced autophagy through FOXO1. (A) The effect of the PI3K/AKT agonist 740 Y‐P and inhibitor LY294002 on the protein levels of p‐FOXO1. (B) The effect of a FOXO1 inhibitor on autophagy‐related protein levels. (C) The effect of a FOXO1 inhibitor on autophagic flux. Scale bars = 20 μm. (D) The effect of the FOXO1 inhibitor on cellular migration. Scale bars = 100 μm. One‐way anova was utilized to compare multiple groups means with one independent variable. Two‐way anova was employed to compare multiple groups means with two independent variables. Post hoc comparisons were conducted using Tukey's *t*‐test. *P vs. PBS, *P < 0.05, ***P < 0.001 and NS means not significant. ^#^ *P* vs. Tp47, ^#^ *P* < 0.05, ^##^ *P* < 0.01, ^###^ *P* < 0.001. Abbreviations: LY, LY294002; PBS, phosphate‐buffered saline.

To investigate whether FOXO1 is involved in the regulation of autophagy by PI3K/AKT, the PI3K/AKT agonist 740 Y‐P and the inhibitor LY294002 were used. After pretreatment with the PI3K/AKT agonist 740 Y‐P, the protein expression of p‐FOXO1 was increased compared with that in the Tp47 group (*p* < 0.001). After pretreatment with the PI3K/AKT inhibitor LY294002, the protein expression of p‐FOXO1 was further decreased compared with that in the Tp47 group (*p* < 0.05; Figure 5A). To further prove the role of FOXO1 in Tp47‐promoted autophagy and migration, cells were pretreated with the FOXO1 inhibitor AS1842856 for 1 h and then incubated with 25 μg/mL Tp47 for 24 h. The FOXO1 inhibitors suppressed the degradation of P62 (*p* < 0.01), the elevation of Beclin‐1 expression (*p* < 0.01), and the conversion of LC3I to LC3II (*p* < 0.01) caused by Tp47 (Figure 5B). To demonstrate whether FOXO1 influenced autophagic flux, the mRFP‐GFP‐LC3 adenovirus was used. In the merged images, the FOXO1 inhibitor AS1842856 increased the number of yellow LC3 spots (representing autophagosomes) (*p* < 0.001), while red spots (representing autolysosomes) showed no significant difference compared with those in the control groups (*p* > 0.05; Figure 5C). These results indicated that autophagic flow could be blocked by the FOXO1 inhibitor AS1842856. Compared with that in the Tp47 group, the number of yellow LC3 spots and red spots was significantly reduced after intervention with the FOXO1 inhibitor AS1842856 (*p* < 0.001; Figure 5C), suggesting that Tp47 induces autophagic flow through FOXO1. The effect of FOXO1 on the migration ability of HMC3 microglia was further detected. Pretreatment with the FOXO1 inhibitor AS1842856 reversed the reduction in cell migration caused by Tp47 (*p* < 0.01; Figure 5D). In brief, PI3K/AKT stimulated HMC3 cell autophagy by promoting FOXO1 nuclear translocation.

## 4 DISCUSSION

Numerous studies have shown that microglial migration plays a key role in regulating central nervous system injury during several infectious diseases, cancers and neurodegenerative disorders.^17^, ^18^ The normal function of microglia is to migrate precisely to sites of neural injury. The role of overactivation of microglia induced by lipopolysaccharide, a prominent cell wall component of gram‐negative bacteria, in promoting migration has been extensively studied.^19^, ^20^ Interestingly, transwell migration assay results showed that the *Treponema pallidum* membrane protein Tp47 inhibited microglial migration, showing an effect opposite that of bacterial lipopolysaccharide. *Treponema pallidum* is known to lack lipopolysaccharides but can express a variety of outer membrane lipoproteins.^21^ These lipoproteins are associated with the tissue adhesion and dissemination of *T. pallidum* and can induce an immune response in the body. *Treponema pallidum* infection elicits a complex immune response in which innate immunity, humoral immunity and cellular immunity all play a role. Therefore, microglia, equivalent to macrophages in the brain and spinal cord, whose migration function is inhibited by the *T. pallidum* outer membrane lipoprotein Tp47, may contribute to *T. pallidum* immune escape, by which *T. pallidum* exerts a survival strategy.

Autophagy and migration are two important biological processes in mammals. Autophagy is closely related to macrophage migration and adhesion. Specifically, autophagy disassembles focal adhesions and regulates their stability, ultimately regulating the migration of cells.^22^ In some tumours and inflammatory diseases, promoting autophagy can induce the level of adhesion and migration of tumour cells and macrophages.^23^ In this study, Tp47 significantly reduced the migration of microglia and increased the level of autophagy. This finding indicates that altering the autophagy level of microglia will affect microglial adhesion and migration. To explore the potential role of Tp47‐induced autophagy in suppressing microglial cell migration, HMC3 cells were treated with autophagy inhibitors 3‐MA and BafA1. Interestingly, the inhibition of autophagy further suppressed the migration ability of HMC3 cells. These results indicate that Tp47‐induced autophagy may promote Tp47‐induced cell migration and the precise mechanisms by which Tp47 ultimately inhibits microglial cell migration deserve further investigation.

Tyrosine kinase receptors are activated by extracellular and intracellular factors to activate PI3K, which catalyses the conversion of the substrate PIP2 to PIP3.^24^ PIP3 works in concert with phosphoinositide‐dependent kinase 1 to fully activate AKT.^25^ Activation of AKT transmits signals to various downstream targets, such as mTOR and FOXO1, promoting the protein synthesis, proliferation and growth of cells; accelerating cell metabolism; and inhibiting autophagy.^26^, ^27^ Our results showed that Tp47 induced autophagy in HMC3 cells by inhibiting the PI3K/AKT pathway. Several studies have shown that the PI3K/AKT signalling pathway plays an important role in the regulation of autophagy.^28^, ^29^ Autophagosome formation is initiated by the PI3P molecule synthesized by PI3K class II kinase, which is usually activated after interaction with Beclin‐1.

FOXO1, a member of the FOXO family, is a direct downstream substrate of the PI3K/AKT signalling pathway.^30^ When the PI3K/AKT pathway is activated under various stimuli, activated AKT phosphorylates FOXO1 and changes the conformation of FOXO1. FOXO1 binds to 14‐3‐3 proteins to block the nuclear localisation signal of FOXO1 by forming dimers and promotes the migration of FOXO1 from the nucleus to the cytoplasm.^31^, ^32^ Phosphorylated FOXO1 is retained in the cytoplasm and exhibits diminished transcription factor activity. In the present study, Tp47 resulted in decreased phosphorylated levels of FOXO1, as well as increased expression and nuclear retention of FOXO1. Furthermore, the PI3K/AKT activator 740 Y‐P significantly inhibited FOXO1 pathway activation, implying that FOXO1 is located downstream of the PI3K/AKT pathway. The FOXO1 inhibitor AS1842856 significantly reversed Tp47‐induced migration and autophagy. These results indicated that PI3K/AKT promoted Tp47‐induced autophagy via FOXO1.

Several possible limitations should be considered. First, the inhibition of microglial migration by Tp47 suggests that further investigations are necessary to ascertain if *T. pallidum* is able to avoid an immune response by this mechanism. Second, the migration of microglia occurs through a complex regulatory network, and this study only revealed the regulation of migration by autophagy. Third, we need to perform in vivo studies to confirm our in vitro findings.

**FIGURE 6:**
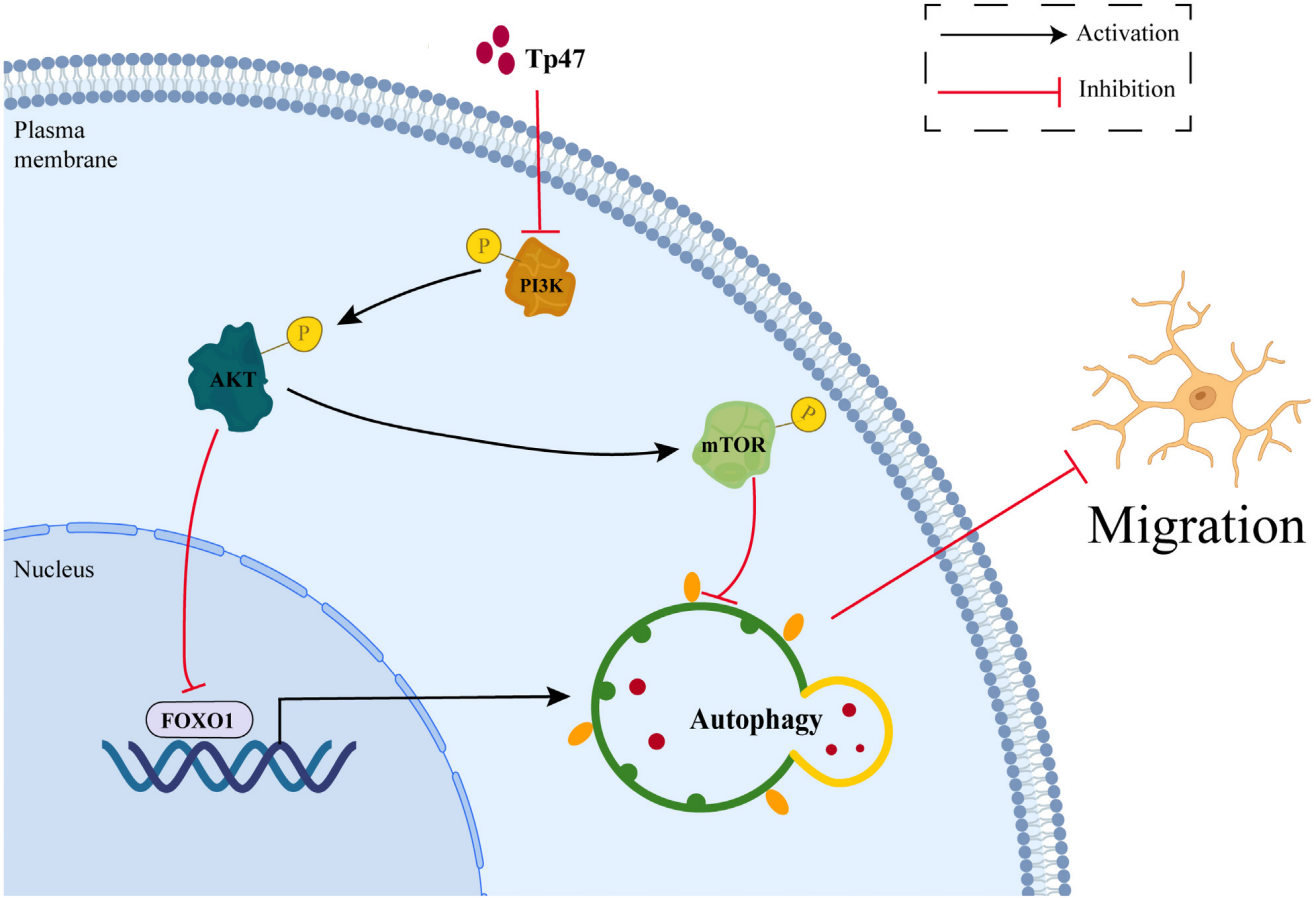
Schematic illustrating that Tp47 induces autophagy in HMC3 cells via the PI3K/AKT/FOXO1 pathway to impair migration.

In conclusion, this study elucidated that Tp47 induced autophagy in HMC3 cells via the PI3K/AKT/FOXO1 pathway to impair migration (Figure 6). These findings add to our knowledge of the immune escape strategy of *T. pallidum* invading the central nervous system.

## AUTHOR CONTRIBUTIONS

**Tian‐Ci Yang:** Funding acquisition (equal); project administration (equal); supervision (equal); writing – review and editing (equal). **Lin Xie:** Data curation (equal); investigation (equal); visualization (equal); writing – original draft (equal). **Wei Li:** Investigation (equal); methodology (equal); visualization (equal); writing – original draft (equal). **Xin‐Qi Zheng:** Investigation (equal); visualization (equal). **Li‐Li Liu:** Funding acquisition (equal); investigation (equal); methodology (equal). **Li‐Rong Lin:** Data curation (equal); investigation (equal). **Jian‐Jun Niu:** Funding acquisition (equal); investigation (equal); supervision (equal).

## FUNDING INFORMATION

This work was supported by the National Natural Science Foundation of China [grant numbers 81973104, 82172331, 81971147, 81972028, 82272370, 82271387], the Key Projects for Province Science and Technology Program of Fujian Province, China [grant number 2020D017, 2019D008], the Natural Science Foundation of Fujian Province, China [grant number 2021J02055], the Fujian provincial health technology project [grant numbers 2020CXB047].

## CONFLICT OF INTEREST STATEMENT

None of the authors have any conflicts of interest to disclose.

## CONSENT FOR PUBLICATION

All authors declared to consent for publication.

## Data Availability

The data supporting the conclusions of this article will be made available by the authors, without undue reservation.
