# Correction to ‘Treponema pallidum membrane protein Tp47 induced autophagy and inhibited cell migration in HMC3 cells via the PI3K/AKT/FOXO1 pathway’

**DOI:** 10.1111/jcmm.70070

**Published:** 2024-09-29

Xie L, Li W, Zheng XQ, et al. Treponema pallidum membrane protein Tp47 induced autophagy and inhibited cell migration in HMC3 cells via the PI3K/AKT/FOXO1 pathway. *J Cell Mol Med*. 2023;27(20):3065‐3074. doi: 10.1111/jcmm.17872

In Xie et al.,^1^ the published article contains errors in Figure 1, Figure 2D, Figure 3E and Figure 5D. This issue occurred because of a mix‐up in the organization of our original data, likely due to the large volume of images involved. The corrected Figure 1, Figure 2, Figure 3 and Figure 5. are below. The authors confirm that the conclusions of this article remain unchanged.

**FIGURE 1:**
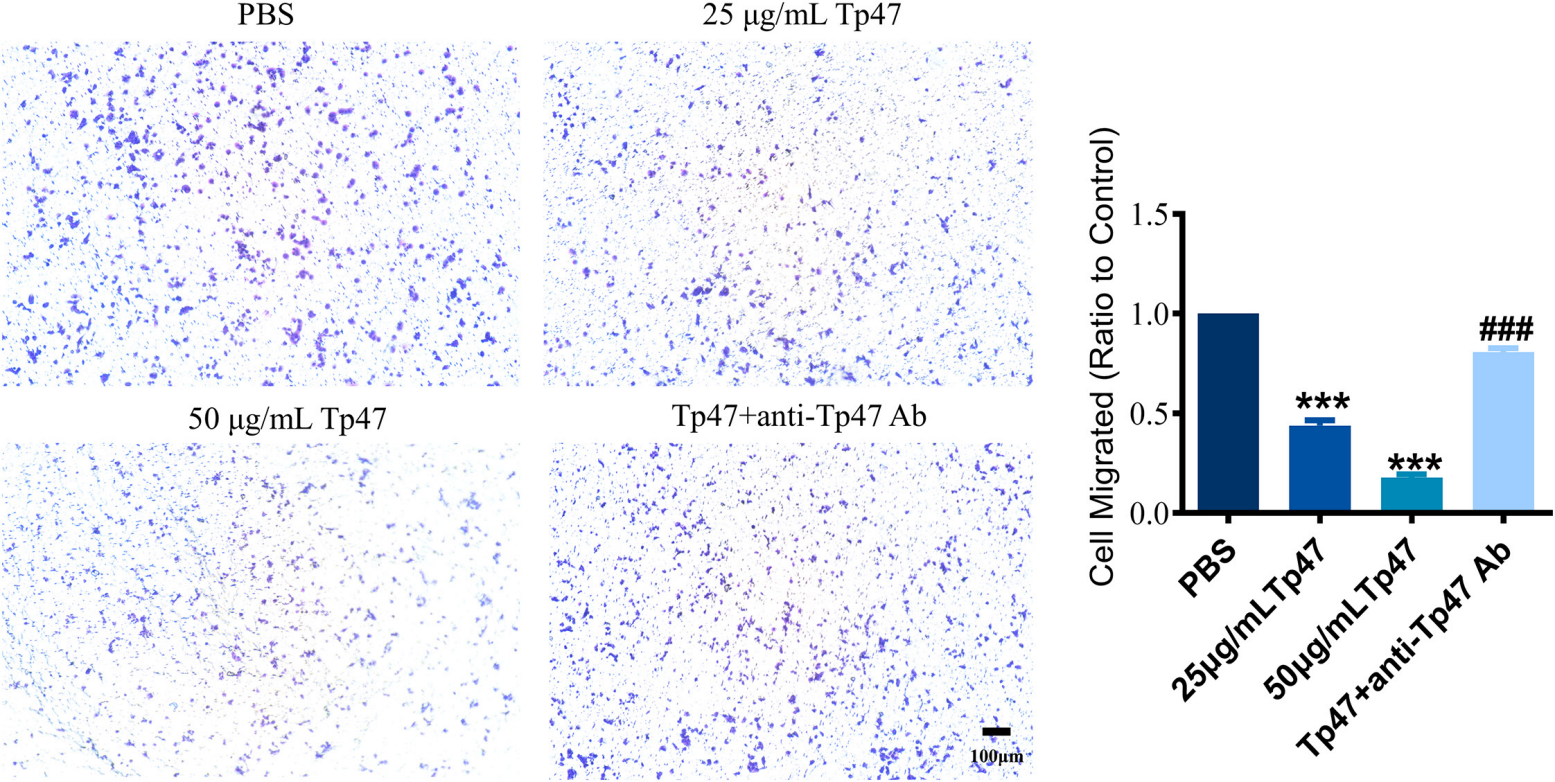


**FIGURE 2:**
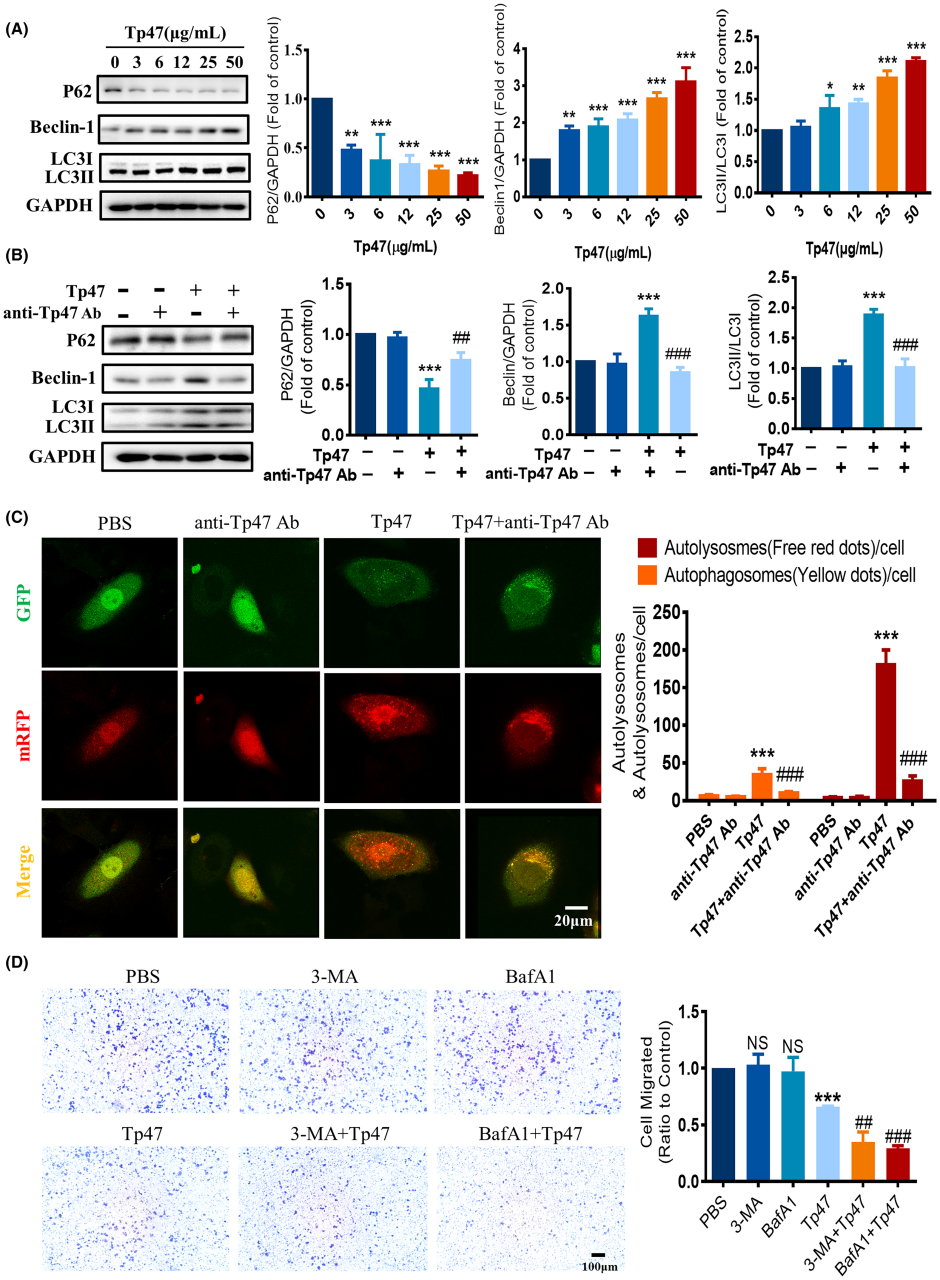


**FIGURE 3:**
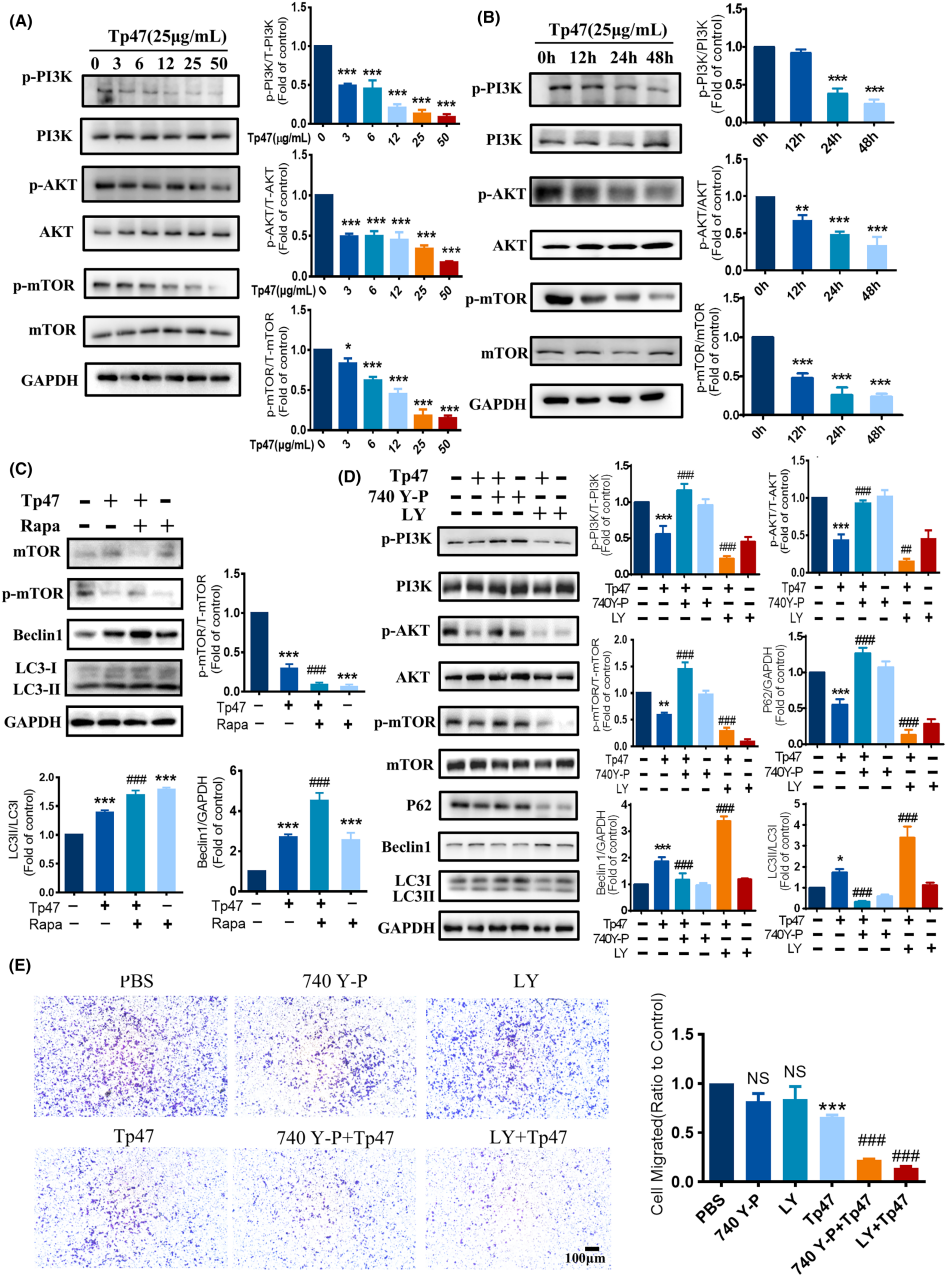


**FIGURE 5:**
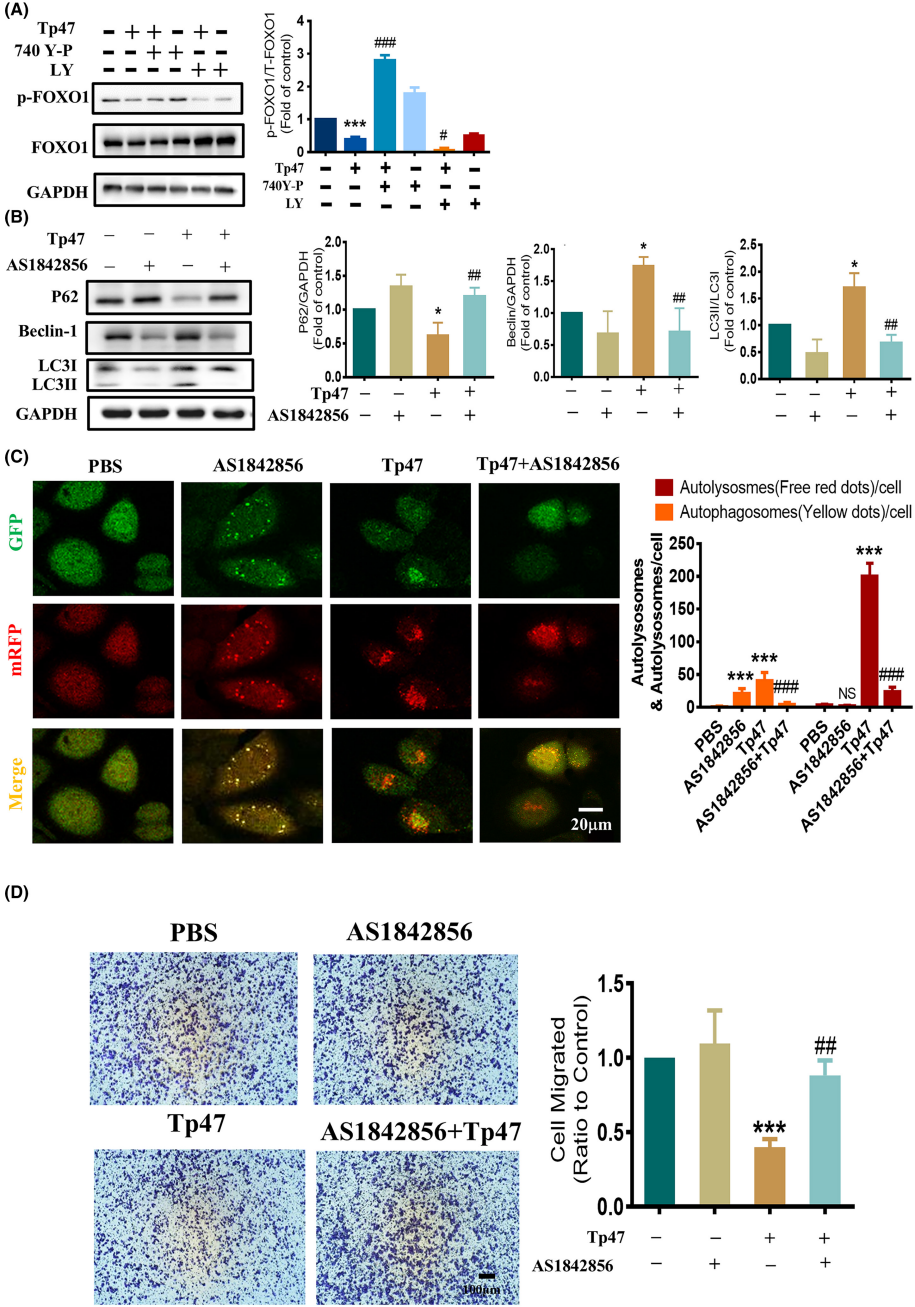


## Supporting information


Figures S1.
