# Supplementary material for: Correction to ‘Treponema pallidum membrane protein Tp47 induced autophagy and inhibited cell migration in HMC3 cells via the PI3K/AKT/FOXO1 pathway’
Source: J Cell Mol Med. 2024 Sep 29;28(18):e70070. doi: 10.1111/jcmm.70070 (PMC12047208; doi:10.1111/jcmm.70070)
Supplement: Supplementary file 1 — Figures S1. [file JCMM-28-e70070-s001.docx]

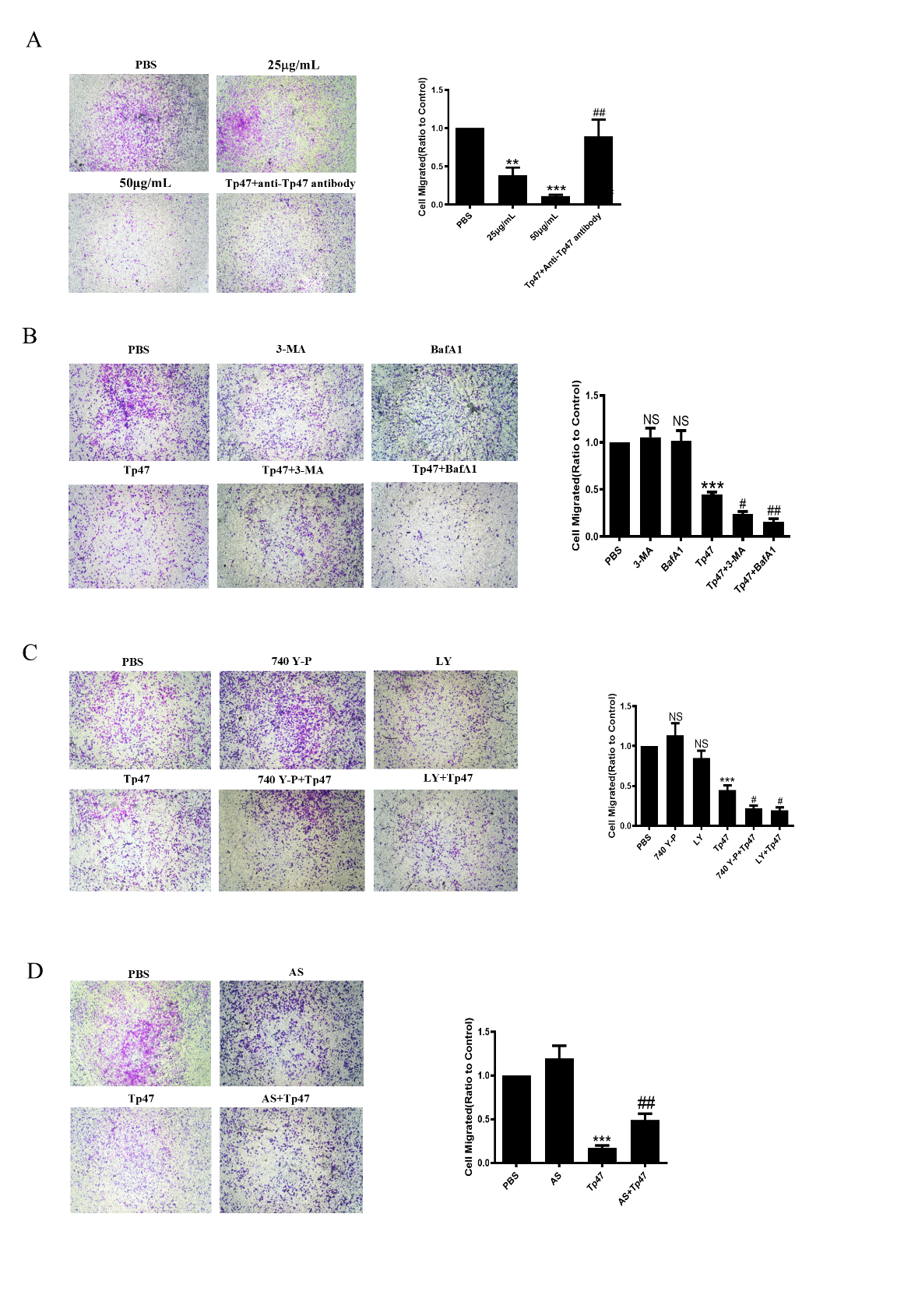


Figure S1. Original images of HMC3 migration (4X). (A) Tp47 impaired HMC3 cell migration. (B) The effect of the autophagy inhibitors 3-MA and BafA1 on HMC3 cell migration. (C) The effect of PI3K/AKT inhibition on HMC3 cell migration. (D) The effect of the FOXO1 inhibitor on cellular migration. *P vs. PBS, *P<0.05, **P<0.01, and ***P<0.001. #P vs. Tp47, ##P < 0.01. Abbreviations: PBS, phosphate-buffered saline; BafA1, bafilomycin A1; 3-MA, 3-methyladenine; anti-Tp47 Ab, anti-Tp47 antibody. LY, LY294002; Rapa, rapamycin.
